# Exploring Shared Stress Experiences Among Teens With Chronic Health Conditions

**DOI:** 10.1111/cch.70258

**Published:** 2026-03-11

**Authors:** Victoria B. Nicksic, Hannah K. Dettinger, Anne D. Letocha

**Affiliations:** ^1^ School of Medicine and Public Health University of Wisconsin‐Madison Madison Wisconsin USA; ^2^ Pauquette Center Madison Wisconsin USA; ^3^ School of Nursing University of Wisconsin‐Madison Madison Wisconsin USA

## Abstract

**Background:**

Adolescents and young adults with chronic health conditions (CHCs) experience significant condition‐related stress in addition to everyday stress. This increases their risks of high cumulative stress, which could affect current and future health and well‐being. Prior research is limited by including participants in different developmental stages, a narrow focus on one or a few conditions, or exploring concepts other than stress. Few studies have focused on the stress experiences of teens with diverse CHCs who are in one developmental stage, including the stressors they experience and their impact on teens' lives more broadly.

**Objective:**

The purpose of this study was to explore the unique stress experiences of teens with CHCs.

**Methods:**

Semi‐structured, audio‐only interviews were conducted with teens with CHCs. Interview questions explored everyday and CHC‐related stressors and teens' responses to these stressors. Data were analysed using reflexive thematic analysis, with qualitative description as the theoretical foundation. Codes, sub‐themes, and themes were identified and defined through an iterative process.

**Results:**

Fifteen teens aged 14–17 years old with a variety of CHCs completed study interviews. Three central themes were generated to reflect teens' condition‐related stress experiences: (1) Living with a CHC; (2) My CHC makes me different; and (3) Response to living with a CHC. Teens' experiences were unique and diverse, highlighting the importance of exploring individual perspectives.

**Conclusion:**

This study broadens the understanding of stress experiences in teens with a variety of CHCs, highlighting how condition‐related stress can permeate multiple aspects of life. Findings underscore the need for individualized assessment of condition‐related stress and the development and implementation of tailored interventions designed to mitigate the impact of condition‐related stress on teens' lives.

## Summary

1. Stress is a unique concept that needs to be assessed separately from other, related concepts in research and clinical practice.
2. Research focused on specific developmental stages, such as middle adolescence, provides insights into experiences that may not be shared with older or younger teens, highlighting the importance of studies of specific developmental stages.
3. Research on teens with chronic health conditions should include categorical and non‐categorical research, which provide distinct and important information on stress and other experiences across and within conditions.
4. Clinicians should pay careful attention to what teens say about their condition‐related stress and what they need to help them manage that stress. All teens are unique, and clinical care should be individualized to honour that.

## 1 Introduction

An increasing number of adolescents and young adults (AYA) in the United States living with chronic health conditions (CHCs) must manage their conditions during a challenging and potentially stressful developmental stage (Black et al. 2024; Brosco et al. 2024; Department of Health and Human Services 2022). Youth experience everyday stressors related to family and peer relationships, school and self‐identity formation (Núñez‐Regueiro and Núñez‐Regueiro 2021). Those with CHCs also experience stressors related to living with and managing a CHC (Delamater et al. 2013; Ersig et al. 2016; Ferro et al. 2016; Ravid et al. 2015; Walklet et al. 2016; Compas et al. 2012; Wagner et al. 2023). This combination puts youth with CHCs at increased risk of high cumulative stress, which could affect current and future health and well‐being (Belkin et al. 2024; Esdaille et al. 2024; Mihaila et al. 2025; Wallander and Varni 1992).

Multiple studies describe potential implications of living with a CHC in children, adolescents and young adults. Although age ranges vary, these studies often include participants who are ages 7–12, 12–18 or 18–30 years, blurring the lines between distinct developmental stages with unique experiences (Delamater et al. 2013; Compas et al. 2012; Chew et al. 2018; Ferro 2014; Eines et al. 2022; Wilson and Stock 2019; Cobham et al. 2020). Many studies also combine age groups (e.g., children and adolescents and AYA) (Chew et al. 2018; Harper et al. 2024; Sligo et al. 2019), making it difficult to determine which stressors are most significant for which age group(s). Teens with CHCs who are 14–17 years old and in the developmental stage of middle adolescence are starting to assume greater responsibility for CHC management as they anticipate the transition to adulthood and out of the family home (Chew et al. 2018; Wilson and Stock 2019; Long et al. 2021; Perrin et al. 2012; Eiser 1993; Lagercrantz et al. 2020; Allen and Waterman 2026). They also experience stress and uncertainty about the future (Ersig et al. 2016).

Other studies have explored specific aspects of living with a CHC (e.g., friendships, subjective well‐being and coping), often using quantitative measures (Delamater et al. 2013; Ferro 2014; Long et al. 2021) (Compas et al. 2012; Chew et al. 2018; Eines et al. 2022; Perrin et al. 2012). Although they did not assess stress exposure or response (Courtwright et al. 2022; James et al. 2024; Määttä et al. 2022), some of these studies provided insights into actual and potential stressors. These include daily condition management, adherence to recommended treatments, condition‐specific stressors (e.g., hypoglycaemia worry in type 1 diabetes), physical symptoms and the impact of CHCs on friendships, social connections and school participation. Teens with CHCs described fitting the condition and its management into their lives as challenging, often due to unpredictability in symptoms and management (Chew et al. 2018; Eines et al. 2022; Harper et al. 2024; Bingemann et al. 2024; Wicks et al. 2018). In other studies, children, adolescents and young adults described difficult diagnostic journeys and delayed diagnosis, often due to limited knowledge and experience of healthcare providers (Harper et al. 2024; Barned et al. 2021; Kirk and Hinton 2019). AYAs with CHCs also felt different from their peers (Wilson and Stock 2019; Sligo et al. 2019; Lagercrantz et al. 2020; Kirk and Hinton 2019; Cardillo 2010) or that they were ‘missing out’ on what their peers were experiencing (Sligo et al. 2019).

Additional research has explored concepts related to stress response in studies of AYAs with CHCs. These studies focused on concepts including identity, loneliness, coping, adaptation, resilience, self‐esteem, emotional or psychosocial well‐being, health‐related quality of life and mental health (Wagner et al. 2023; Chew et al. 2018; Wilson and Stock 2019; Sligo et al. 2019; Wicks et al. 2018; Lewis et al. 2024; Ersig et al. 2025). However, they did not explore potential relationships between these concepts and stress exposure or response. Few studies have asked teens directly about their condition‐related stressors (Ersig et al. 2016; Ersig 2019).

Finally, many studies have taken a categorical (i.e., condition‐specific) approach to studies of the experiences of AYAs with CHCs, focusing on one or a few conditions (Ersig et al. 2016; Chew et al. 2018; Sligo et al. 2019; Lagercrantz et al. 2020; Barned et al. 2021; Ersig 2019; Karadag et al. 2025). These studies often report findings specific to included CHCs; for example, teens with IBD described stressors related to GI symptoms and medications required to control their conditions (Barned et al. 2021). However, taken together, the results from these studies identified stress exposures and responses that may be shared across conditions. A noncategorical approach (Stein and Jessop 1989) would provide important insights and improve the limited knowledge of stress experiences shared by teens with diverse CHCs.

This study aimed to fill gaps in knowledge of shared stress experiences in teens with a variety of CHCs who are all in the developmental stage of middle adolescence using a semi‐structured interview guide. Previous studies have included participants with a wide range of ages that span unique development stages, focused on one or a few conditions, explored concepts other than stress and the lived experiences of teens with CHCs and described condition‐specific stress experiences. However, few studies have explored teens' stress experiences more broadly. As a result, knowledge of the stress experiences of middle adolescents with CHCs, including the stressors they are exposed to and how they perceive and respond to those stressors, is limited.

## 2 Purpose and Study Aim

This cross‐sectional qualitative descriptive study was part of a larger, mixed‐methods study that compared cumulative stress exposure and psychological and physiological stress response between teens with and without CHCs. The purpose of this study was to explore the unique stress experiences of teens with CHCs. This manuscript presents data from interviews of 15 teens 14–17 years old with a CHC. We focused on teens 14–17 years old to ensure that they were in a similar developmental stage. Results from the other study aims and parents' interviews will be reported separately.

## 3 Methods

### 3.1 Inclusion and Exclusion Criteria

Eligible teens (1) were diagnosed at least 1 year prior to study enrollment with a chronic physical health condition (CHC), such as asthma, diabetes, bone, joint or muscle disorders, food allergies or other similar conditions; (2) had internet access; and (3) self‐identified as English speakers and readers. Exclusion criteria included cognitive or developmental disability, a CHC diagnosis within the last year or a self‐reported diagnosis of a severe mental health condition (e.g., bipolar disorder).

### 3.2 Sample Identification and Enrollment

Ethical approval, including a waiver of signed consent, was obtained from the Univeristy of Wisconsin‐Madison Minimal Risk IRB (IRB #2022‐1316). Participants were recruited using letters sent to parents or legal guardians of potentially eligible teens who participated in the Survey of the Health of Wisconsin (SHOW) or received care at the local healthcare system, mass emails sent to a university listserv, digital or printed study information posted in clinics and community sites and school newsletters. Because eligible teens were under 18 years of age, recruitment materials targeted parents or legal guardians.

Study recruitment materials included brief study information and a QR code that linked to a study information survey. Those who were interested in participating completed the survey and provided contact information for the teen's parent or legal guardian. A study team member contacted those who were interested to schedule a phone call to confirm eligibility and complete the informed consent process. During the phone call, a study team member provided detailed information on study procedures, confirmed the teen's eligibility, obtained verbal permission from the parent/legal guardian for the teen to participate in the parent study and obtained the teen's verbal informed assent. After permission and assent for the parent study were obtained, the study team member shared information on optional study interviews with teens with CHCs and their parents/legal guardians. A separate verbal permission/assent process was completed with those interested in participating in study interviews. Interviews were scheduled for a separate date and time. Teens who completed the study interview received a $15 electronic gift card as compensation for their time.

### 3.3 Data Collection

Semistructured audio‐only interviews were conducted from March to September 2023 using Webex, a secure, HIPAA‐compliant platform (Webex by Cisco 2025). All interviews were audio‐recorded with participant permission. The average duration of the interviews was approximately 30 min (range, 15–60 min). The interview guide was developed based on the transdisciplinary model of stress and the senior author's prior studies (Ersig et al. 2016; Ersig 2019; Ersig and Williams 2018; Epel et al. 2018). Initial questions focused on teens' day‐to‐day lives, including everyday stressors. Subsequent questions were used to ask about condition‐related stressors, including what was most stressful, changes in stressors over time and response to stress experiences. Study interviews were transcribed verbatim and then reviewed and verified by study team members.

### 3.4 Analysis

The theoretical foundation for this analysis is qualitative description, guided by a constructivist philosophy (Doyle et al. 2019). Teens were considered experts in their own experiences, and interview questions were designed to obtain information about their unique, subjective experiences (Doyle et al. 2019; Bradshaw et al. 2017). A foundational tenet of qualitative description as applied in this study is that reality is multiple and subjective and exists within individual contexts (Doyle et al. 2019; Bradshaw et al. 2017). This philosophical orientation was appropriate for achieving the study aim, as we sought to understand teens' unique experiences of stress related to living with a CHC.

Two analysts (ADL, VBNinitials blinded) completed a reflexive thematic analysis of the transcribed text (Braun and Clarke 2020; Braun et al. 2022) using Microsoft Word and Microsoft Excel (La Pelle 2004). Analysis included the following phases: (1) becoming familiar with the data; (2) coding; (3) generating initial themes; (4) developing and reviewing themes; (5) refining, defining and naming themes, which included identification of sub‐themes; and (6) writing the report of the data analysis and results (Braun and Clarke 2025). Conflicts were resolved through discussion.

## 4 Reflexivity

Reflexive thematic analysis requires that researchers describe how their backgrounds and experiences might influence their approach to data analysis (Braun et al. 2022; Braun and Clarke 2024). The senior author (ADL) is a nurse researcher whose research and clinical career have focused on individuals with or at risk for CHCs and their family members. Previous studies explored stress exposure, stress response and quality of life in children, adolescents and young adults diagnosed with CHCs. At the time of analysis, the first author (VBN) was a paediatric endocrine fellow and a board‐certified paediatrician with clinical expertise in paediatric medical care who had managed numerous patients, including those with chronic illnesses. Her clinical role informed the analysis through her engagement in the practical realities of paediatric healthcare. The second author (HKD) was a social work student with extensive experience doing clinical interviews as a case manager. She conducted most of the study interviews.

## 5 Results

Fifteen teens (mean age, 15.67 years; range, 14–17 years) with a variety of CHCs completed study interviews. Seven participants had asthma/food allergies, three had neurologic conditions, two had gastrointestinal conditions, and three reported multiple conditions. Eight were assigned female sex at birth. To maintain confidentiality, additional demographic data are not provided.

**FIGURE 1:**
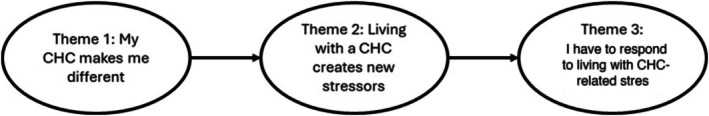
The figure outlines the relationship among the three themes. Specifically, teens feel different because of their CHC diagnoses. Living with their CHCs creates new stressors, and they must then respond to living with CHC‐related stress.

Three central themes were generated from teens' narratives about their condition‐related stress experiences: (1) My CHC makes me different, (2) Living with a CHC creates new stressors, and (3) Response to living with CHC‐related stress. The themes have a linear relationship: Being diagnosed with a CHC makes a teen different from their peers, which leads to a life that includes new CHC‐related stressors, to which teens have to respond (Figure 1 and Table 1).

**TABLE 1:** Definitions of themes and sub‐themes.

Themes and sub‐themes	Definitions
**Theme 1: My CHC makes me different**	**Having a CHC diagnosis makes me different from my peers; I experience things they do not**.
Sub‐theme 1: Being different	My CHC makes me different from everyone else, and it's noticeable to me and other people.
Sub‐theme 2: Social impact	My CHC influences my relationships and connections with other people.
**Theme 2: Living with a CHC creates new stressors**	**My CHC diagnosis creates new stressors, changing the way I live my life**.
Sub‐theme 1: Daily impact	In my daily life, I experience stress from CHC‐related symptoms, managing my condition and its impact on my schedule.
Sub‐theme 2: This is never going away	My CHC is never going away, and I worry about what is to come.
Sub‐theme 3: Disease journey	It's taken me a long time to get where I am today, and I do not know exactly what the future will bring.
Sub‐theme 4: Struggling to be believed	I do not feel like my experiences are fully acknowledged or understood.
Sub‐theme 5: Context is important	My CHC‐related stress is influenced by my current situation, including my physical and social environments.
**Theme 3: Response to living with CHC‐related stress**	**When I experience CHC‐related stress, this is what I do in response**.
Sub‐theme 1: Staying on high alert	I always need to be prepared and aware/alert.
Sub‐theme 2: Safe places	I seek out certain situations, or with certain people, where I feel safe.
Sub‐theme 3: I'm learning how to live with my CHC	I'm learning about my CHC, how to manage my CHC‐related stress and find purpose in my diagnosis.

### 5.1 Theme 1: My CHC Makes Me Different

Teens described a pervasive sense of feeling different from their peers due to their CHC, which is reflected in the theme *My CHC makes me different*. These physical, social or emotional differences often interfered with their ability to participate fully in what they perceived as ‘normal’ teenage life. Two sub‐themes captured this experience: (1) being different and (2) social impact. ‘Being different’ was often framed in terms of not feeling ‘normal’: ‘I feel like I have this voice in the back of my head telling me that I'm not normal’ (Teen 5). Another teen shared, ‘I feel excluded from the normal teenage life because I have to think about stuff so much more and I can't just go with the flow like a lot of other people’ (Teen 6). The ‘social impact’ sub‐theme includes teens' descriptions of how their perceived differences affected their relationships with others. Some teens described how their conditions caused stress for those around them, such as family members: ‘I know that it's stressful (for others) because, obviously, no one wants to see their kid or their friend or anyone they know … be in so much pain’ (Teen 11). Teens also reported changed relationships, with one teen sharing that ‘the way they [my parents] act towards me has changed a lot since I was diagnosed’ (Teen 2). For some teens, others' perceptions of their conditions did not align with the teen's reality: ‘some of my friends have made comments to me over the years that lets me know that they don't think my disease is that big of a deal because they don't understand it, and they don't take the time to understand it’ (Teen 5).

### 5.2 Theme 2: Living With a CHC Creates New Stressors

The theme *Living with a CHC creates new stressors* reflects how a CHC diagnosis influenced teens' lives and how they anticipate it will affect their lives in the future. This theme had five sub‐themes: (1) context is important, (2) daily impact, (3) disease journey, (4) struggling to be believed and (5) this is never going away.

The first sub‐theme, ‘context is important’, included teens' descriptions of how stress related to their CHC changes based on their current situation, including their physical environment and the people they are with. As one teen put it, ‘I definitely think it's easier to feel less stressed when you're around people that are similar to you’. The same teen shared that ‘… being at people's houses or restaurants can be stressful because I can't check the food label for the food that I'm eating because it wasn't made or bought by me’ (Teen 14).

The second sub‐theme, ‘daily impact’, focused on how a CHC affects teens' daily lives, including their routines and activities: ‘The most stressful thing for me is probably working my life around my disease’ (Teen 5). This sub‐theme also included teens' descriptions of their CHC‐related symptoms: ‘the constant feeling of the fact that my body doesn't work correctly and it doesn't work well’ (Teen 13).

Teens also shared how stress related to their CHCs changes over time, often in unexpected ways, experiences captured in the third sub‐theme, ‘disease journey’. This included the often lengthy and difficult diagnostic process as well as changes in symptoms. One teen first reflected on the diagnostic process, stating that ‘the waiting period for getting (a) diagnosis was obviously difficult’, then went on to share that ‘the main thing is that it's just so unpredictable … I describe it as a game of whack a mole’ (Teen 11).

The fourth sub‐theme, ‘struggling to be believed’, reflected teens' feelings that their experiences are often dismissed or misunderstood, leading to frustration or invalidation, especially when interacting with healthcare providers or the healthcare system. One teen speculated that ‘most of us could have been diagnosed at a much younger age if there was less for lack of better word, medical gaslighting going on’ (Teen 9). For some, this led to self‐doubt: ‘I get in my head and I'm like well what if I really just have is nothing, which is like not the case because I'm having health issues. But I have just kind of gotten to a point where no one's been able to give us much of anything. I just gaslit myself’ (Teen 11). Outside of the healthcare system, other teens noted experiences of misplaced trust, with one sharing that ‘[O]ne place where the food I got was supposed to be safe, but clearly it wasn't’ (Teen 4) after an unexpected exposure to an allergen.

The fifth and final sub‐theme for *living with a CHC creates new stressors*, ‘this is never going away’, highlighted teens' realizations that their CHCs are lifelong, leading some to worry about the future and the long‐term impact of the condition on their lives. ‘Sometimes I'll think about it, and I'll be like, whoa, this is forever, it's not just gonna stop one day’ (Teen 6). Another teen lamented the fact that they were never going to be ‘healthy’, stating, ‘… I was born with it, and I'll die with it, and it's hard to know that you'll never be a healthy person’ (Teen 13).

### 5.3 Theme 3: Response to Living With CHC‐ Related Stress

The final theme, *Response to living with CHC‐ related stress*, included three sub‐themes that reflect how teens respond to CHC‐related stress: (1) staying on high alert, (2) safe places and (3) I'm learning how to live with my CHC.

The sub‐theme ‘staying on high alert’ demonstrated the need for constant awareness. Some teens described their need to be constantly vigilant to anticipate and head off potential crisis situations related to their CHC. This was particularly relevant for teens with food allergies, such as one who shared that they ‘… check the same food again and again’ (Teen 7). Another emphasized the importance of preparing ahead of time to minimize the impact of their CHC, stating, ‘if you're not careful about things, things could get worse, so just always have your medicine with you’ (Teen 4). Other teens described how staying on high alert in uncertain situations could lead to additional stress, such as one who shared, ‘just being unsure about what I'm eating and the ingredients can really make me stressed out’ (Teen 14).

In contrast, ‘safe places’ were situations or relationships where teens felt secure and safe from challenges experienced in other contexts. Teens described times, places and people that they sought out to give them the opportunity to lower their guard and experience relief from CHC‐related stress: ‘I definitely have a better safe feeling at home because I'm sure that what I'm eating or what I'm around is safe and I definitely feel more at ease and less stressed’ (Teen 14). Another teen described how some of their friends who also have chronic conditions are safe places for them: ‘I do have friends that have other, more chronic conditions, that have a better idea of how that can impair even if it's not related to my situation’ (Teen 9).

In the final sub‐theme, ‘I'm learning how to live with my CHC’, teens described the process of learning about the condition, managing CHC‐related stress and, for some, finding a sense of purpose in their diagnosis. ‘Just trying to ride the waves of the pain, like validating the fact that it hurts so bad and then I just have to get through it and that it's gonna suck. It does suck but I've been through it before, and I can get through it again’ (Teen 11). For some teens, learning how to live with their CHC also included practices of self‐awareness, advocacy, emotional regulation and acceptance: ‘… even though stress is a big part of my life, I feel grateful almost to my disease for showing me how to be empathetic and how to relate to people's pain on a greater scale than I feel others can …’ (Teen 5). This teen also stated, ‘it is important to advocate for yourself and you need to understand what your body is going through to be able to show people what you need and ask for the help’. Yet, the degree of acceptance varied between teens, with another stating, ‘I kind of just have to live with it I guess’ (Teen 3).

## 6 Discussion

This study explored the stress experiences of teens with a variety of chronic health conditions, revealing how condition‐related stress permeates their lives and has the potential to contribute to cumulative stress over time (Slopen et al. 2018). Our study findings are unique because they focus on stress experiences, rather than other related concepts. We identified shared stress experiences of middle adolescents with different CHCs using a qualitative data collection tool, ensuring conceptual consistency and clarity. Prior research has examined concepts related to stress, such as coping or loneliness, focused on one or a few conditions or included children and/or young adults, in addition to teens (Delamater et al. 2013; Compas et al. 2012; Ferro 2014; Eines et al. 2022; Wilson and Stock 2019; Cobham et al. 2020; Lagercrantz et al. 2020). Previous studies examined stress related to type 1 diabetes (Eines et al. 2022; Ersig 2019), IBD (Barned et al. 2021), food allergies (Bingemann et al. 2024; Ersig and Williams 2018) and other individual conditions. One study explored stress in teens with two contrasting diseases, asthma and cancer (Sligo et al. 2019), although research that focuses on teens with a variety of CHCs remains rare. Studies taking a noncategorical approach often include young adults, whose experiences may differ from those of teens (Wilson and Stock 2019; Harper et al. 2024; Sligo et al. 2019; Wicks et al. 2018).

Teens conveyed the realities of living with a CHC over time, describing their diagnostic journeys, their lives now and their worries for the future. Many teens described experiencing CHC‐related symptoms before receiving a specific diagnosis. When the diagnostic journey was prolonged, teens felt that their experiences were not fully understood or taken seriously, especially by healthcare providers. Some even described feeling gaslit, as reported in studies of young adults with CHCs (Harper et al. 2024; Bugwadia et al. 2024). Feeling unheard or unseen sometimes led teens to question themselves and their own experiences. For some, these experiences occurred just as they were beginning their lifelong relationship with the healthcare system and could affect their willingness to engage with the system and receive needed care in the future.

Teens also spoke about the need to adhere to routines and manage ongoing symptoms, consistent with prior research (Chew et al. 2018; Eines et al. 2022; Bugwadia et al. 2024; Apple 2017). Multiple participants described how stress varied depending on their current context. Unfamiliar environments or new situations had a significant impact on teens, even for relatively common experiences, such as eating at a new restaurant. Although new experiences are often thought of as exciting or spontaneous, they may also generate stress for teens with CHCs that require careful management and adherence to condition‐related restrictions.

Although stress experiences are shared across conditions, results from this study demonstrate that they can manifest differently in different CHCs. For example, some teens with food allergies described experiences of misplaced trust that occurred when eating at a trusted restaurant and being unexpectedly exposed to allergens. Data from the Food Allergy Research & Education's (FARE) Patient Registry noted that over 50% of allergic reactions in restaurants occur despite informing restaurant staff of an allergy (Oriel et al. 2021; Golden et al. 2024). Initially positive experiences with a restaurant may lead teens with food allergies to trust that a restaurant is safe; however, this trust can be broken when unexpected exposures and reactions occur, putting teens' safety and their lives at greater risk. Others describe similar experiences of not being heard by healthcare providers, especially during the journey to their CHC diagnosis. These incidents seemed especially disheartening for teens and added new complexity to life with a CHC. Although our aim was to identify stress experiences shared across conditions, these results indicate that some experiences may be unique to certain CHCs. Additional insights into CHC‐related stress variability between conditions and among teens with the same diagnosis could be further explored in mixed‐methods studies that integrate qualitative and quantitative data. Future studies should take categorical and noncategorial approaches with a variety of CHCs to further explore variability and factors contributing to variability in stress experiences within and across conditions. This would provide insight into how shared stress experiences contribute to cumulative stress exposure. Exploring the unique circumstances or situations in which these shared experiences manifest could enable future categorical research to primarily focus on CHC‐specific findings.

Condition‐related stress was described as both unpredictable and persistent. Teens experienced constantly fluctuating daily stress in the context of a persistent, unrelenting chronic condition. Teens' stress was not just focused on the present. Descriptions of stress experiences included anxiety about the future and permanence of disease‐related stress, which echo findings from previous research (Chew et al. 2018; Wilson and Stock 2019; Cobham et al. 2020; Lagercrantz et al. 2020). The CHC and related stress affect teens' daily routines, choices and long‐term plans. Future longitudinal studies could explore stress experiences over time and how they align with developmental stages; results could be used to develop interventions that could be adapted and adjusted over time to meet current developmental needs.

Teens also shared how they felt different from their peers in multiple domains, including their daily lives and routines, their interactions with others and how they think others perceive them, similar to findings from previous studies (Wilson and Stock 2019; Sligo et al. 2019; Lagercrantz et al. 2020; Kirk and Hinton 2019; Cardillo 2010). Many teens described needing to be constantly aware of how their CHC could affect their participation in physical and social activities that are viewed as part of ‘normal’ teenage life. Experiences of feeling different also shaped teens' relationships with others and, for some, led to concern for how their condition impacts those around them. Teens were acutely aware of the stress their CHC causes others, particularly parents, and described how this seemed to alter others' perceptions of them. Pinquart (Pinquart 2013) noted similar dynamics, highlighting that some parents find it challenging to balance parental warmth and overprotection with demandingness after a CHC diagnosis. Comparing the stress experience of teens with CHCs and their parents could provide insight into the impact of CHC‐related stress on familial relationships.

Finally, teens described personal strategies for navigating life with a CHC, including taking breaks, creating space from a specific stressor, learning more about their conditions, practicing acceptance, and engaging in self‐advocacy. Others identified safe places where they could temporarily let down their guard, as described in other studies of well‐being in teens with CHCs (Courtwright et al. 2022). These strategies helped balance persistent uncertainty and the need to remain constantly vigilant.

The results of this study prompted reflection on how healthcare providers can support teens living with CHCs during middle adolescence, a developmental stage with multiple changes in different life domains (Allen and Waterman 2026). Teens in this study shared how their CHCs and related stress experiences affect all areas of their lives. Clinicians caring for teens with CHCs should listen closely to what teens say they need. Developing and implementing targeted interventions that directly address teens' concerns could help teens manage the stress that comes with living with a CHC while starting to think about the transition to adulthood and out of the family home, as well as the process of transferring their healthcare from paediatric to adult healthcare systems. Interventions could include formalized support systems, peer networks or access to trusted safe spaces where teens can find relief from the stress of their conditions. Future studies could also compare CHC‐related stress and the timeliness of healthcare transition and transfer of care.

This small cross‐sectional study has limitations. Because of funding and time constraints, we were limited to 15 study interviews. Future studies should explore stress experiences in larger groups of teens with a variety of CHCs to ensure that findings represent the wide range of teens living with CHCs. Teens self‐selected into the interview phase of the study; thus, results may reflect the experiences of those who were ready and willing to share those experiences. Future studies could consider alternative, accessible approaches to interview‐based data collection, such as open‐ended online questions. Most interviews were also completed during the summer, which could have altered teens' stress experiences. Yet, this study also has strengths, including its focus on teens 14–17 years old with a variety of CHCs.

## 7 Conclusions

This study fills an important gap in knowledge of the types of stress experiences of teens with a variety of CHCs. Results highlight the potential for future interventions to be implemented across subspecialties.

## Author Contributions

**Victoria B. Nicksic:** formal analysis, methodology, validation, visualization, writing – original draft, writing – review and editing. **Hannah K. Dettinger:** investigation, writing – review and editing. **Anne D. Letocha:** conceptualization, investigation, funding acquisition, writing – original draft, methodology, validation, visualization, writing – review and editing, formal analysis, project administration, data curation, supervision, resources.

## Funding

Victoria Nicksic was supported by National Institutes of Health T32DK077586‐10. Research support was provided to Anne D. Letocha by a Research and Scholarship Committee Award from the School of Nursing, UW‐Madison and the UW‐Madison Office of the Vice Chancellor for Research with funding from the Wisconsin Alumni Research Foundation. The project described was also supported by the Clinical and Translational Science Award (CTSA) program, through the NIH National Center for Advancing Translational Sciences (NCATS), grant UL1TR002373. The content is solely the responsibility of the authors and does not necessarily represent the official views of the NIH. Research reported in this publication was supported by the Real‐World Evidence to Advance Community Health (REACH Program). The REACH Program is funded by federal grants (70%) and by nongovernmental sources (30%). The program receives funding from the National Institute of Environmental Health Sciences (R24ES036005) and the National Cancer Institute (P30CA014520) of the National Institutes of Health. The content of this publication is solely the responsibility of the authors and does not necessarily represent the official views of the National Institutes of Health. The authors would like to thank the University of Wisconsin‐Madison School of Medicine and Public Health as well as the study participants for their contributions.

## Ethics Statement

This study was approved by the University of Wisconsin‐Madison Minimal Risk IRB (IRB # 2022‐1316) and included a waiver of signed consent.

## Consent

Verbal permission for the teen's participation was obtained from the parent/legal guardian, and verbal assent was obtained from the teen.

## Conflicts of Interest

The authors declare no conflicts of interest.

## Data Availability

Because of the need to maintain participant confidentiality, supporting data are not available.
